# A Cheap Substitute for a Vapour-Bath

**Published:** 1846-08

**Authors:** 


*A Cheap Substitute for a Vapour-Bath*.—Dr. Serre (d'Alais) recommends the following means of inducing abundant transpiration. "Take a piece of lime about half the size of your fist, and wrap around it a wet cloth, sufficiently wrung to prevent water running from it. A dry cloth is to be several times wrapped around this. Place one of these packets on each side of the patient when in bed. An abundant humid heat is soon developed by the combination of the lime with the water, which quickly induces copious transpiration—the effect of the apparatus lasting for two hours at least. When sweating is fully etablished, we may withdraw the lime, which is now reduced to a powder, and is easily removed. In this way, neither copious drinks nor loading the bed with coverings is required."—*Gazette Medicale.*

